# Molecular Characterization of *Wolbachia* Endosymbionts and Their Association With Canine Dirofilariasis in Colombo District, Sri Lanka

**DOI:** 10.1155/japr/9996613

**Published:** 2026-06-20

**Authors:** Neyon Loku Gamage, Koshila Ranasinghe, Wasana Rodrigo

**Affiliations:** ^1^ Department of Zoology and Environmental Management, Faculty of Science, University of Kelaniya, Sri Lanka, kln.ac.lk; ^2^ Department of Zoology, Open University of Sri Lanka, Sri Lanka, ou.ac.lk

**Keywords:** canine dirofilariasis, *Dirofilaria immitis*, PCR-based detection, sequence, *Wolbachia*

## Abstract

Canine dirofilariasis is caused by *Dirofilaria*, a type of filarial parasite that can also infect humans and is becoming a growing concern in Sri Lanka. Previous studies have noted that Sri Lanka has some of the highest numbers of dirofilariasis cases in Asia. This study was aimed at detecting *Wolbachia* bacteria—which are found inside *Dirofilaria* parasites—using molecular methods and also at identifying which *Dirofilaria* species are present in dogs in the Colombo District, Sri Lanka. Blood was collected from 368 dogs of various breeds between May and December 2025. Of these, only 35 samples (9.51%) tested positive for microfilaria using a microscope. These samples were then analyzed using DNA extraction and PCR with both general *Dirofilaria* and species‐specific primers. At the same time, samples were also screened for *Wolbachia*, a bacterium that is important for *Dirofilaria* survival and reproduction, using *Wolbachia*‐specific primers (wsp). Of the 35 microfilaria‐positive samples, 18 (51.43%) were positive for *Dirofilaria*, and of those 18, only three (16.67%) were positive for *Wolbachia*. Among the *Dirofilaria*‐positive samples, one (5.56%) was identified as *Dirofilaria immitis*, 11 (61.11%) as *Dirofilaria repens*, and three (16.67%) as *Dirofilaria asiatica*; a few samples did not match any of the species‐specific primers. The PCR products for both *Dirofilaria* and *Wolbachia* were verified by gel electrophoresis and sequencing. The sequence results showed the presence of *D. repens* and *D. asiatica* in dogs. Importantly, this is the first molecular evidence of *D. immitis* in Sri Lanka and the first molecular identification of *Wolbachia* in *Dirofilaria* species in the country. The study also found that both *D. repens* and *D. immitis* can infect the same dog. These findings provide new information about canine dirofilariasis in Sri Lanka and highlight the need for *Wolbachia*‐targeted parasite control and accurate molecular diagnosis to guide treatment and control efforts.

## 1. Introduction

Dirofilariasis is a zoonotic disease caused by nematodes of the *Dirofilaria* genus, which belongs to the Onchocercidae family [1]. Mainly, these nematodes can be seen in various mammalian species, dominantly in dogs and less in cats and humans [2–4]. There are about 40 recognized species of *Dirofilaria*, transmitted by mosquito species. *Dirofilaria immitis*, *Dirofilaria repens*, *Dirofilaria asiatica*, *Dirofilaria tenuis*, *Dirofilaria ursi*, and *Dirofilaria spectans* are some of the species that infest humans [5, 6].

Marker genes can be used as the species‐specific targets for the PCR‐based methods, which can be used to differentiate and sensitize between *D. immitis*, *D. repens*, and *D. asiatica*. Mitochondrial, nuclear, and symbiont‐associated genes have been used as specific generic markers over the past years in order to identify these parasites′ closely related species and clinical diagnoses [7]. Among these mitochondrial genes, cox1 is highly informative due to its relatively high mutation rate, maternal inheritance, and less recombination, making it particularly suitable for the identification of closely related species of *Dirofilaria* [8, 9]. In the present study, the cox1 gene was used for the molecular detection of *Dirofilaria* species, as it provides high resolution for species‐level identification. In Sri Lanka, molecular methods have revealed that a significant amount of the filarial infections were related to the *D. asiatica* [10], but there are no reported cases of *D. immitis* [11]. The use of traditional diagnostic methods, such as microscopy and antigen detection, has lower sensitivity and specificity for identifying and differentiating species of microfilariae, leading to misidentification and underreporting of circulating species [12]. Because of these difficulties in differentiating the *Dirofilaria* spp., a problematic situation may arise when there are multiple species coexisting or when the density of the microfilaria is low, which cannot be detected by other traditional methods [13].

PCR‐based approaches offer high sensitivity and specificity, enabling the detection of infections even at lower parasitic levels or in asymptomatic animals. For example, a molecular characterization study that took place in Turkey has demonstrated the presence of multiple vector‐borne pathogens in canine populations while highlighting their epidemiological role as reservoir hosts [14]. Similarly, a previous study conducted in Iran had highlighted the widespread occurrence of *D. immitis* in canine populations, identifying the importance of dogs in disease transmission cycles using molecular‐based approaches [15]. Molecular detection has also proven effective in the identification of other filarial parasites, such as *Babesia bigemina* in the rural dog population, while ensuring the reliability of PCR‐based tools [16]. Collectively, these studies have proven the importance of molecular‐based approaches in the surveillance of canine vector‐borne diseases and the detection of different filarial species.

*Dirofilaria* spp. may carry a bacterium called “*Wolbachia*” endosymbiont DNA [17]. In recent years, much attention has turned toward the bacterial partner of filarial nematodes: *Wolbachia*. These *Wolbachia* are intracellular alphaproteobacteria that show endosymbiosis with a broad range of nematodes as well as arthropods [18]. The filarial nematodes store these bacteria in their intracytoplasmic vacuoles within the hypodermal lateral cords of the female and male nematodes and in the female reproductive systems/organs [19]. *Wolbachia* are transmitted through the eggs of hosts and change the host biology in different ways, such as manipulations of reproductive systems, such as feminization, parthenogenesis, killing males, and incompatibility of sperm and eggs in arthropod hosts [20]. However, in filarial nematodes, *Wolbachia* exists as an obligate mutualistic endosymbiont that is important for the development of parasites, embryogenesis, fertility, and survival, making it a potential target for antifilarial therapies [21, 22].

For the detection and characterization of *Wolbachia* endosymbionts, the wsp gene was used in the present study. The wsp gene enables differentiation among *Wolbachia* strains and supergroups compared with other conserved genes, such as 16S rDNA [20, 23]. Because of this high variability, it makes wsp a suitable marker for strain typing and phylogenetic clustering of *Wolbachia* associated with filarial nematodes.

Molecular and parasitological studies have shown that canine dirofilariasis infections are widely distributed across multiple provinces, with prevalence rates between approximately 30% and over 60% in different provinces, particularly within the Western Province, including Colombo and Negombo districts ([11, 24]. Although detailed district‐specific prevalence data for the Colombo District is limited, available studies suggest that canine dirofilariasis remains consistently present across urban and semiurban communities [11]. In addition, the specific data on *Wolbachia* prevalence in canine dirofilarial infections in the Colombo district, as well as in the whole country, remains limited [25].

Globally, the endosymbiosis between *Wolbachia* and *Dirofilaria* has been well reported, but in Sri Lanka, there are no reports on their symbiosis [22, 26] that could aid in controlling strategies for *Dirofilaria* parasites. In particular, gaps remain in understanding the simultaneous detection of parasite‐symbiont systems in naturally infected dogs. Therefore, the current study addressed these gaps by applying molecular tools to improve the detection and identification of *Dirofilaria* species using the mitochondrial cox1 gene, to detect and characterize the association between *Wolbachia* and *Dirofilaria* species using the wsp gene, to assess the coinfection of different *Dirofilaria* species in the same host, and to explore potential breed‐associated patterns of infection within the sampled canine population in Colombo District, Sri Lanka.

## 2. Methodology

### 2.1. Collection of Dog Blood Samples

**Figure 1:**
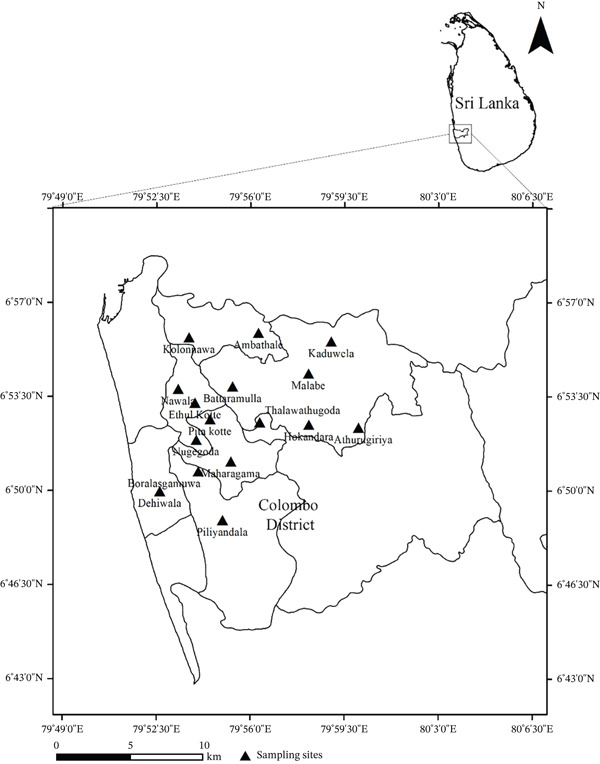
Canine blood sampling sites in Colombo District, Sri Lanka.

The Colombo District was selected in this study for screening *Dirofilaria* parasites in dog blood based on the prevalence of dirofilariasis cases reported [26]. Blood samples were collected from different breeds, including both client‐owned and free‐roaming dogs, representing a range of breeds (German shepherd, Labrador, golden retriever, Rottweiler, local breed, crossbred, etc.) based on a convenience sampling approach, based on accessibility and owner consent during the study period, through the veterinarian services of pet clinics in the Colombo District (Figure 1) from May to December 2025. Dogs were sampled irrespective of sex (F, 196; M, 172) and age, and both symptomatic (*n* = 327) and asymptomatic (*n* = 41) individuals were included. For owned animals, informed consent was obtained from the owners prior to sample collection, while sampling of stray dogs was conducted with institutional ethical guidelines and animal handling protocols with the help of veterinarians. A total of 368 canines were included in the study. The distribution of sampled dogs across breeds was as follows: bully type, 46; Labrador, 69; Rottweiler, 67; crossbreed, 113; beagle, 6; German shepherd, 22; ridgeback, 8; golden retriever, 19; stray dog, 9; Pomeranian, 2; dachshund, 2; shih tzu, 3; and cocker spaniel, 2. Blood samples were placed in EDTA tubes, and the samples were stored at 4°C until further processing. Ethical approval was obtained from the Institute of Biology (IOB), Sri Lanka, under ERC IOBSL 413/03/2025.

### 2.2. Staining of Dog Blood With Leishman Stain

A small amount of blood sample (2 *μ*L) was taken from the EDTA tube and added to a clean slide, and a thin blood smear was made on the slide. Then, the smear was allowed to air dry completely. The entire slide was covered with a prepared Leishman stain and kept for 4 min. The slide was allowed to air dry completely. After the slide had dried, it was observed under a light microscope.

### 2.3. Knott′s Test for the Canine Blood Samples

One milliliter of the blood sample was mixed with 2% formalin (9 mL) in a centrifuge tube. Then the tube was inverted several times in order to mix, ensuring the blood was fully lysed. The mixture was centrifuged at 1400 rpm for about 5 min. Then the supernatant was carefully removed while leaving only the sediment at the bottom. Next, two drops of 1% methylene blue were added to the tube to stain any microfilariae present. Finally, a drop of the mixture was placed on the slide and then covered with a coverslip and examined under a light microscope.

Both of the identification methods were done in order to get better results.

### 2.4. Extraction of Genomic DNA From Dog Blood Using the Spin Method

The collected blood samples were subjected to extraction using a QIAamp spin‐column method. First, proteinase K (20 *μ*L) was pipetted into 1.5 mL microcentrifuge tubes. Dog blood samples (200 *μ*L) were added to the same microcentrifuge tubes. Buffer AL (200 *μ*L) was added to each sample, then it was mixed by vortexing for 15 s. Next, the prepared samples were incubated at 56°C overnight. Then, the overnight‐incubated microcentrifuge tubes were centrifuged at 1000 rpm for 1 min to remove any drops from the lid. At the same time, Buffer AE was incubated at 56°C until it was further used. Absolute ethanol (200 *μ*L) was added to each tube and again mixed by vortexing for 15 s. After mixing, the microcentrifuge tubes were centrifuged at 1000 rpm for 1 min to remove any drops from the lid. Next, each mixture was added to QIAamp minispin columns without wetting the rim. Then, the columns with 2 mL collection tubes were centrifuged at 8000 rpm for 1 min. While discarding the collection tubes with the filtrate, the columns were placed on clean 2 mL collection tubes, which were provided. Again, without wetting the rim, to each QIAamp minicolumn, Buffer AW1 (500 *μ*L) was added. Next, the columns were centrifuged at 8000 rpm for 1 min. While discarding the collection tubes with the filtrate, the columns were again placed on clean 2 mL collection tubes, which were provided. Without wetting the rim, to each QIAamp minispin column, Buffer AW2 (500 *μ*L) was added. The columns were centrifuged at 14,000 rpm for 3 min.

Each QIAamp minispin column was placed in a new 2 mL collection tube while discarding the old collection tubes with the filtrates. Then again, the columns were centrifuged at 14,000 rpm for 1 min. Next, the collection tubes were discarded with the filtrate. The QIAamp minispin columns were placed on 1.5 mL microcentrifuge tubes carefully, and then the columns were incubated with Buffer AE (200 *μ*L). Each column was incubated at room temperature for 1 min. Then, the columns were centrifuged at 8000 rpm for 1 min. Next, the columns were discarded, and the 1.5 mL microcentrifuge tubes with the eluates were stored at −20°C until further processing. Detection of the *Wolbachia* endosymbiont was also done with the same extracted samples with specific primers. DNA concentration and purity were detected using the NanoDrop method.

### 2.5. Optimization of PCR Conditions for *Dirofilaria* spp., *D. immitis*, *D. repens*, and *Wolbachia*

After the reconstitution of primers, the PCR conditions of the species were optimized. To prepare one master mixture solution (20 *μ*L), GoTaq Green Master Mix (12.5 *μ*L), forward primer (5 *μ*M, 0.8 *μ*L), reverse primer (5 *μ*M, 0.8 *μ*L), and PCR water (5.9 *μ*L) were added together. For the negative sample, along with the master mixture, an extra 5 *μ*L of PCR water was added, whereas to the other samples, 5 *μ*L of DNA template was added.

For *Dirofilaria* spp.: Amplification of PCR was performed using primers that were designed to amplify the 219 bp region of mitochondrial cytochrome oxidase subunit I gene of *Dirofilaria* spp. parasite [2]. Sequences of the primers were as follows: forward: DR COI‐F1 (5 ^′^‐AGT GTT GAT GGT CAA CCT GAA TTA‐3 ^′^) and reverse: DR COI‐R1 (5 ^′^‐GCC AAA ACA GGA ACA GAT AAA ACT‐3 ^′^). Twenty microliters of master mixture and 5 *μ*L of DNA template were subjected to the PCR for initial denaturation at 95°C for 3 min, followed by 35 cycles of denaturation at 95°C for 30 s, annealing at 57°C for 30 s, and extension at 72°C for 1 min. The final extension was at 72°C for 1 min.

For *D. immitis*: Amplification of PCR was performed using primers that were designed to amplify the 300 bp region of the mitochondrial cytochrome oxidase subunit I gene of *Dirofilaria* spp. parasite [27]. Sequences of the primers were as follows: forward: DI COI‐F1: (5 ^′^AGTGTAGAGGGTCAGCCTGAGTTA‐3 ^′^) and reverse: DI COI‐R1: 5 ^′^ACAGGCACTGACAATACCAAT‐3 ^′^). Twenty microliters of master mixture and 5 *μ*L DNA template were subjected to the PCR for initial denaturation at 94°C for 5 min, followed by 35 cycles of denaturation at 94°C for 30 s, annealing at 57°C for 45 s, and extension at 72°C for 1 min. The final extension was at 72°C for 7 min.

For *D. repens*: Amplification of PCR was performed using primers that were designed to amplify the 750 bp region of the mitochondrial cytochrome oxidase subunit I gene of *Dirofilaria* spp. parasite [27]. Sequences of the primers were as follows: forward: (Diro‐cox1‐F): 5 ^′^‐GCTTTGTCTTTTTGGTTTACTTTT‐3 ^′^) and reverse: (Diro‐cox1‐R): 5 ^′^‐TCAAACCTCCAATAGTAAAAAGAA‐3 ^′^). Twenty microliters of master mixture and 5 *μ*L DNA template were subjected to the PCR for initial denaturation at 94°C for 4 min, followed by 35 cycles of denaturation at 94°C for 30 s, annealing at 57°C for 45 s, and extension at 72°C for 1 min. The final extension was at 72°C for 7 min.

For *Wolbachia* sp.: The *Wolbachia* surface protein gene was amplified using the designed *wsp* primer to amplify the 600 bp region of the gene of *Wolbachia* bacteria [28]. Sequences of the primers were as follows: forward: 81 primer: 5 ^′^TGGTCCAATAAGTGATGAAGAAACTAGCTA‐3 ^′^ and reverse: 691 primer: 5 ^′^AAAAATTAAACGCTACTCCAGCTTCTGCAC‐3 ^′^. Twenty microliters of master mixture and 5 *μ*L of DNA template were subjected to the PCR for initial denaturation at 94°C for 2 min, followed by 35 cycles of denaturation at 94°C for 30 s, annealing at 55°C for 45 s, and extension at 72°C for 1 min. The final extension was at 72°C for 10 min.

PCR products were analyzed by electrophoresis in 1.5% agarose gel prepared in 1X TBE buffer. The gel was stained with ethidium bromide (0.5 *μ*g/mL) and visualized under a UV transilluminator. A 1000 bp DNA ladder was used as a molecular size marker to estimate amplicon sizes.

### 2.6. DNA Sequencing

Specific unpurified PCR products were sent to Macrogen company (Macrogen Korea 10F, 254 Beotkkot‐ro, Geumcheon‐gu, Seoul 08511, Rep. of Korea) to perform further characterization by sequence analysis using bidirectional Sanger sequencing. Raw chromatograms were inspected and trimmed to remove low‐quality bases using the BioEdit tool (Version 7.2) through sequence alignment and analysis. Assembled full sequences were then subjected to the NCBI nucleotide BLAST tool (http://www.ncbi.nlm.nih.gov/BLAST). The search set was configured with standard database parameters for choosing the sequences that shared 98%–100% homology with complete DNA sequences, high query coverage (> 95%), and lower *E*‐values approaching about 0.0 found in the GenBank database. The accession numbers for the sequences obtained from the present study were obtained by submissions to the GenBank database.

For phylogenetic analysis, representative reference sequences of *Dirofilaria* spp. and *Wolbachia* strains were retrieved from GenBank. The evolutionary relationships were identified using the neighbor‐joining method, and the analysis of evolution was conducted in MEGA 12 software using ClustalW. The evolutionary distances, which were computed using the Tamura–Nei and the Tamura‐3 parameters, were used to create the phylogenetic tree, and the node support was assessed using bootstrap analysis with about 92%. All sequences generated in this study were aligned with closely related references to confirm species identity and evolutionary relationships.

## 3. Results

### 3.1. Identification of Microfilaria in Dog Blood Samples

**Figure 2:**
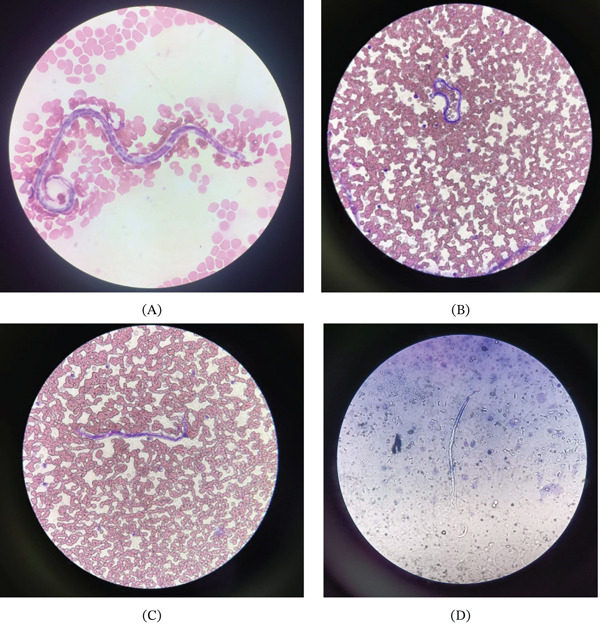
Microscopic view of microfilaria in dog blood samples stained using Leishman stain: (A) x1000 magnification and (B, C) x400 magnification. (D) Microscopic view of microfilaria in dog blood samples tested using Knott′s test (x400 magnification).

The direct dog blood smear, which was stained using Leishman′s stain, showed the presence of microfilaria of nematode worms. After examination through a microscope, the microfilariae appeared as long, slender, thread‐like organisms. The head and tail morphology was clearly visible; the head was curved, whereas the tail was straight and pointed (Figure 2).

A total of 368 blood samples were collected and initially screened by microscopic examination, of which 35 samples were found to be positive for microfilariae. These microscopy‐positive samples were subsequently subjected to PCR analysis, and 18 samples were confirmed as PCR‐positive for *Dirofilaria* spp. Thus, PCR analysis was performed on microscopy‐positive samples only, rather than the entire sample set. The highest percentage for the presence of *Dirofilaria* was detected in Labrador (0.0082%). Both German shepherds and stray dogs (0.0054%) showed similar positive results, whereas Rottweilers, crossbreeds, beagles, ridgebacks, and golden retrievers (0.0027%) showed equal susceptibility for infection (Table 1). Then, the statistical analysis was done in order to identify if there is a relationship between the infection rate and the canine breed.

**Table 1:** Dog breeds with a percentage positive for infection with *Dirofilaria* worms.

Dog breed	Positive number of samples for infection with *Dirofilaria* spp.	Uninfected dog count	Age of the dog for positive samples (years)	Percentage positive from the total samples (*n* = 368)
Bully type	3	43	3	0.0082
			5	
			12	
Labrador	6	63	7	0.016
			10	
			6	
			6	
			8	
			5	
Rottweiler	1	66	8	0.0027
Crossbreed	1	112	8	0.0027
Beagle	1	5	6	0.0027
German shepherd	2	20	6	0.0054
			9	
Ridgeback	1	7	4	0.0027
Golden retriever	1	18	10	0.0027
Stray dog	2	7	5	0.0054
			8	

Table 1 shows the distribution of infection status among different dog breeds. A chi‐square test of independence was performed to determine whether there was a significant association between dog breed and infection status. The results showed a statistically significant association between the breed and infection status, as the *p* value < 0.05, indicating that the infection rates differed significantly among dog breeds.

### 3.2. Molecular Detection of *Dirofilaria* Species With *Dirofilaria-*Specific Primer

#### 3.2.1. DNA Concentration and Purity Using the NanoDrop Spectrophotometer

NanoDrop spectrophotometers confirmed the purity of proteins and nucleic acids using ultraviolet‐visible (UV‐Vis) absorbance for the DNA in dog blood samples. DNA has high purity when the nanodrop ratio is approximately 1.8. Then all samples were within the acceptable range, confirming qualified samples for PCR (Table 2).

**Table 2:** DNA concentration and DNA/protein ratio in DNA extractions.

Sample name	DNA concentration (ng/*μ*L)	Purity
V (bandog/bully type)	41.840	1.94
X (Labrador)	39.340	1.92
A2 (Rottweiler)	23.737	1.8
B2 (crossbreed)	35.473	1.94
C2 (pit bull)	45.920	1.94
D2 (Labrador)	12.556	1.9
E2 (bully type)	16.078	1.83
F2 (Labrador)	19.654	1.88
G2 (Labrador)	54.650	1.73
I2 (Labrador)	39.946	1.79
K2 (beagle)	6.529	1.56
L2 (ridgeback)	21.677	1.78
M2 (Labrador)	16.892	1.91
O2 (stray dog)	23.551	1.9
Q2 (German shepherd)	13.551	1.94
R2 (golden retriever)	8.845	2.02
S2 (stray dog)	17.544	1.88
T2 (German shepherd)	12.293	1.86

The generally accepted range for DNA purity in PCR is an A260/A280 ratio of 1.8–2.0 [29]. According to Table 2 results, the sample (K2), which had lower purity, was rejected and was not subjected to the PCR process.

The PCR products yielded bands, approximately 200–300 bp (Figure 2). The positive bands in the gel image confirmed the presence of *Dirofilaria* parasites in the blood of several dog breeds by the PCR amplification. Forward and reverse complement sequences were aligned with ClustalW multiple alignment, and the full sequence was constructed using the BioEdit tool, and BLAST output of the analyzed sequences A2‐G2 was given as *D. repens* (Table 3).

**Table 3:** BLAST output of analyzed sequences from *Dirofilaria* parasites.

Sample	Accession number received from NCBI	BLAST output best hit
A2	PX945284	*Dirofilaria repens* isolate DCO1 cytochrome oxidase subunit 1 gene, partial cds; mitochondrial (best hit with Sequence ID: KT588609.1)
B2	PX956944	*Dirofilaria repens* isolate DCO1 cytochrome oxidase subunit 1 gene, partial cds; mitochondrial (best hit with Sequence ID: KT588609.1)
C2	PX945286	*Dirofilaria repens* isolate DCO1 cytochrome oxidase subunit 1 gene, partial cds; mitochondrial (best hit with Sequence ID: KT588609.1)
D2	PX945287	*Dirofilaria repens* isolate DCO1 cytochrome oxidase subunit 1 gene, partial cds; mitochondrial (best hit with Sequence ID: KT588609.1)
E2	PX945291	*Dirofilaria repens* isolate DCO1 cytochrome oxidase subunit 1 gene, partial cds; mitochondrial (best hit with Sequence ID: KT588609.1)
F2	PX956943	*Dirofilaria repens* isolate DCO1 cytochrome oxidase subunit 1 gene, partial cds; mitochondrial (best hit with Sequence ID: KT588609.1)
G2	PX945292	*Dirofilaria repens* isolate DCO1 cytochrome oxidase subunit 1 gene, partial cds; mitochondrial (best hit with Sequence ID: KT588609.1)
L2	PX956945	*Dirofilaria repens* isolate DCO1 cytochrome oxidase subunit 1 gene, partial cds; mitochondrial (best hit with Sequence ID: KT588609.1)
M2	PX945298	*Dirofilaria repens* isolate DCO1 cytochrome oxidase subunit 1 gene, partial cds; mitochondrial (best hit with Sequence ID: KT588609.1)
O2	PX945300	*Dirofilaria repens* isolate DCO1 cytochrome oxidase subunit 1 gene, partial cds; mitochondrial (best hit with Sequence ID: KT588609.1)
Q2	PX945299	*Dirofilaria repens* isolate DCO1 cytochrome oxidase subunit 1 gene, partial cds; mitochondrial (best hit with Sequence ID: KT588609.1)
R2	PX945302	*Dirofilaria* sp. *hongkongensis* isolate Chonampara, Thiruvananthapuram, Kerala, voucher VCRC‐BSD29 (mosquito isolate [*Culex* sp.]) cytochrome c oxidase subunit I (COX1) gene, partial cds; mitochondrial (best hit with Sequence ID: PQ222623.1)
S2	PX945303	*Dirofilaria* sp. *hongkongensis* isolate Chonampara, Thiruvananthapuram, Kerala, voucher VCRC‐BSD29 (mosquito isolate [*Culex* sp.]) cytochrome c oxidase subunit I (COX1) gene, partial cds; mitochondrial (best hit with Sequence ID: PQ222623.1)
T2	PX945304	*Dirofilaria* sp. *hongkongensis* isolate Chonampara, Thiruvananthapuram, Kerala, voucher VCRC‐BSD29 (mosquito isolate [*Culex* sp.]) cytochrome c oxidase subunit I (COX1) gene, partial cds; mitochondrial (best hit with Sequence ID: PQ222623.1)

#### 3.2.2. Molecular Detection of *D. immitis* With Species‐Specific Primers

**Figure 3:**
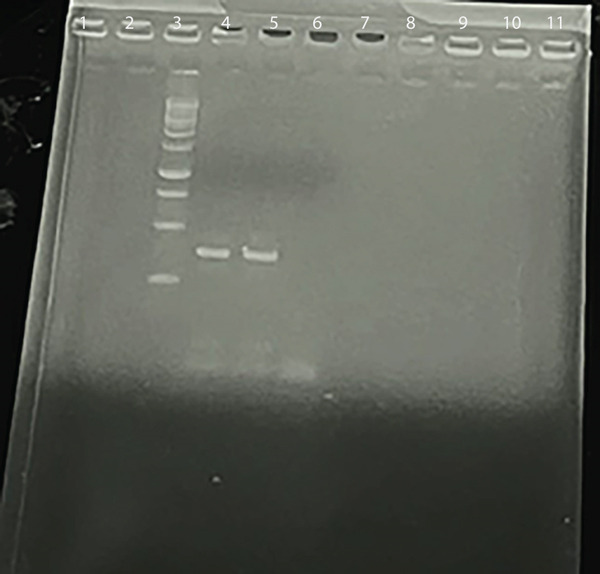
Gel image of PCR products (Lanes 1 and 2: empty; Lane 3: 1000 bp DNA ladder [G5711]; Lanes 4 and 5: positive for *D. immitis* in the dog blood sample [replicate of the same sample]; Lane 6: negative control).

There was only one band in the gel image as positive for *D. immitis* from all samples (Figure 3 and Table 4).

**Table 4:** Dog breeds positive for infection with *Dirofilaria immitis*.

Dog breed	Positive number of samples	Age of the dog (years)	Percentage of positive for infection with *D. immitis* (%)
Ridgeback (IL2) (*n* = 1)	1	4	0.0027

The PCR products from the extracted DNA from dog blood nematodes in different dog breeds yielded bands, approximately 300–350 bp (Figure 3). The positive bands in the gel image confirmed the presence of *D. immitis* in the dog blood by the PCR amplification. The bands in the agarose gel electrophoresis were of good quality and visible, with better separation to send for sequencing.

Forward and reverse complement sequences were aligned with ClustalW multiple alignment, and the full sequence was constructed using the BioEdit tool for the positive sample (Table 5).

**Table 5:** Sequence analysis of the sample positive for *D. immitis*.

Sample	Sequence type	Sequence
IL2	Forward	>H260113‐016_C01_IL2_IFP.ab1 401
		CTATTTTGTAGAGTCTGTGGTTACATACTGTAGGTATTGGTTCTTTGTTGGGTGCTATTAATTTTATAGTTACTACTCATAATATACGTTCTACTGCTGTTACTTTAGATCAAATTAGTATGTTTGTTTGAACTTCTTATTTGACTTCTTTTCTTTTGGTTTTATCTGTTCCTGTTTTGGCTGGTTCTTTGTTGTTTTTATTGTTGGATCGTAATTTTAATACTTCTTTTTATGATACTAAAAAGGGGGGTAATCCTTTATTGTATCAACATTTGTTTTGATTTTTTGGTCATCCTGAGGTTTATGTTATTATTTTACCTGTTATTGGTATTGTCACATACCTGAAATTACTACATATTCGATAGCTGACGGTGCGGCGGTCTGCCAAAAAAAAAAAAAAT
	Reverse	>H260113‐016_A01_IL2_IRP.ab1 391
		GGGGGCGTGCGGAAAAAAATATAGAGTTTGTGCCTCATATATCCCCCCCTCAGGAGATAAAATAAAAAATTTTTCTTTTATATAATATGGAGAGCGTCATTATTTTATATCTAAATATTCCCATACTAATTATATTAACAGCGACAGAGGTTTTTCTCTCACGAACCCCCCCCTCTTTAAGAGTAAAAAGAGAGACACCCCCGGGGCCCCCCCACTCAAACTTTCTTTTTTATATCGTGGGTTTTTTACTGAGACGTGTACAATTGGTGTAGCTCATCATATTTTTTCACCTGTAGTTGTTGAAAAAACAATTATTAATAATTATTTAGTAGATGAAAAATTCATTATTCATAAGAAAAAAGACGACTATGCGGTCGAAAAAACAAAAAAA
	Complete (Accession Number: PX945309)	>Query IL2
		GGGTCAGCCTGAGTTATCTTTAGATAGTATAATTTTGGGATTACATACTGTAGGTATTGGTTCTTTGTTGGGTGCTATTAATTTTATAGTTACTACTCAGAATATACGTTCTACTGCTGTTACTTTAGATCAAATTAGTATGTTTGTTTGAACTTCTTATTTGACTTCTTTTCTTTTGGTTTTATCTGTTCCTGTTTTGGCTGGTTCTTTGTTGTTTTTATTGTTGGATCGTAATTTTAATACTTCTTTTTATGATACTAAAAAGGGGGGTAATCCTTTATTGTATCAACATTTGTTTTGATTTTTTGGTCATCCTGAGGTTTATGTTATTATTTTACCT

#### 3.2.3. Phylogenetic Analysis of *D. immitis* Recorded From the Study

**Figure 4:**
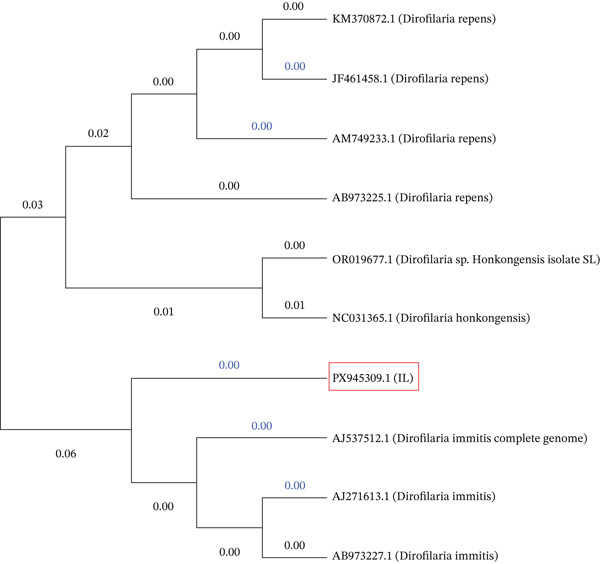
The phylogenetic relationships of the query sequence of *Dirofilaria immitis* with reference sequences in the biological databases.

The phylogenetic analysis revealed that the query sequence of the *D. immitis* (PX945309) isolate from the present study showed considerable homology and proximity to the *D. immitis* complete mitochondrial genome (Accession Number: AJ537512.1) (Figure 4). The common ancestors were represented by the branch points, and genetic distances were shown by the numerical value on each branch.

#### 3.2.4. Molecular Detection of *Wolbachia* Endosymbiosis With *Dirofilaria* Species

Only four samples were positive for the presence of DNA with the *Wolbachia* endosymbiont out of 18 samples, which were positive for *Dirofilaria* spp. German shepherds, ridgebacks, golden retrievers, and Labradors were positive for the *Wolbachia*–*Dirofilaria* endosymbiosis (Table 6).

**Table 6:** Dog breeds with a percentage positive for *Wolbachia*.

Dog breed	Positive number of samples	Age of the dog (years)	Percentage of positive for *Wolbachia* (%)
Ridgeback (L2)	1	4	5.55
German shepherd (T2)	1	6	5.55
Golden retriever (R2)	1	10	5.55
Labrador (G2)	1	10	5.55

PCR samples yielded bands, approximately at 600–650 bp. Through the PCR amplification, the positive bands in the gel image confirmed the presence of *Wolbachia* in the extracted dog blood samples. As the bands in the gel electrophoresis were of good quality and sharp, they were sent for the sequencing process for further analysis to identify the species (Table 7).

**Table 7:** BLAST output of analyzed sequences from *Wolbachia*.

Sample	Accession number received from NCBI	BLAST output best hit
WL2	PX935691	*Wolbachia* endosymbionts of *Paratrechina longicornis* isolate 9 surface protein gene, partial cds (best hit with Sequence ID: KU527461.1)
WR2	PX935692	*Wolbachia* endosymbiont of *Brugia pahangi* isolate FR3 chromosome, complete genome (best hit with Sequence ID: CP050521.1)

#### 3.2.5. Phylogenetic Analysis of *Wolbachia* sp. Recorded From the Study

**Figure 5:**
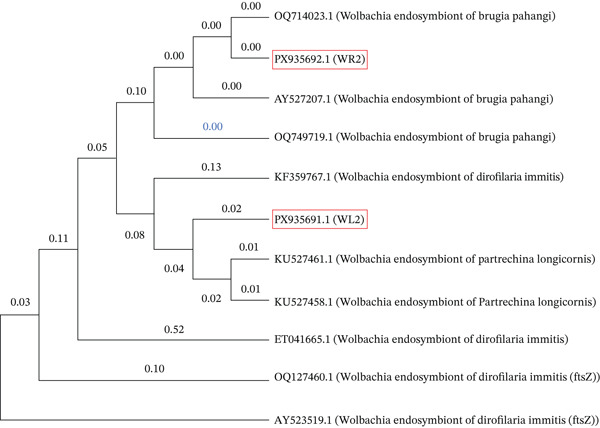
The phylogenetic relationships of the query sequence of *Wolbachia* bacteria with reference sequences in the biological database.

The phylogenetic analysis revealed that the query sequence of the *Wolbachia* bacteria isolate from the L2 sample (WL2) in the present study showed considerable homology and proximity to the *Wolbachia* endosymbiont of the *Paratrechina longicornis* isolate 9 surface protein gene, partial cds (Sequence ID: KU527461.1), and the WR2 sample showed homology to the *Wolbachia* endosymbiont of the *Brugia pahangi* isolate FR3 chromosome, complete genome (Sequence ID: OQ714023.1) (Figure 5). The common ancestors were represented by the branch points, and genetic distances were shown by the numerical value on each branch.

## 4. Discussion

The present study was performed to investigate *Wolbachia* endosymbiosis in *Dirofilaria* spp. through molecular detection, while molecularly identifying the different infecting types of *Dirofilaria* spp., and to assess the possible coexistence of distinct *Dirofilaria* species within a single host. Study reveals first Sri Lankan records on the presence of *D. immitis* in the tested blood samples and the endosymbiosis of *Wolbachia*–*Dirofilaria* species. This provides valuable information on ongoing transmission dynamics within the canine population in the study area. The results indicated the presence of *D. immitis* in samples with a very low percentage abundance. Moreover, the present study findings revealed the coexistence of multiple *Dirofilaria* species within the same host. This result provides molecular evidence of mixed dirofilarial infections, which can suggest repeated exposure to infective mosquito vectors carrying different dirofilarial species [30]. *Wolbachia* DNA was also found in the same ridgeback blood sample in which *D. immitis* was found, proving the endosymbiosis between *Wolbachia* and *D. immitis*.

The chi‐square test demonstrated a statistically significant association between dog breed and infection status (*x* ^2^ = 9.415, *p* < 0.05), indicating that infection prevalence varies among different dog breeds. However, the presence of low expected counts in several canine breeds suggests that these findings have to be interpreted with caution, as small sample sizes can reduce the robustness of chi‐square analyses [31]. Even though higher infection rates were observed in stray dogs and certain other breeds, such as beagles and ridgeback, these observations are likely influenced by limited sample sizes and may not sometimes accurately reflect the true biological susceptibility. According to the present study, stray dogs are the most susceptible to the infection. This may be due to the frequent exposure to the vectors, lack of veterinary care, increased outdoor activities, and absence of proper treatments, making them more vulnerable to the *Dirofilaria* infection [6]. Among owned breeds, Labradors and German shepherds showed a moderate number of infections, which may be attributed to environmental exposure and inconsistent preventive practices rather than inherent breed susceptibility. In contrast, a lower amount of infection rate was observed in crossbred dogs, suggesting the potential advantage of the association of genetic diversity, which may enhance disease resistance in the animal population [32]. Overall, the variation in infection prevalence across breeds appears to be driven more by ecological and management‐related factors than by genetic predisposition alone, highlighting the importance of considering cofounding variables such as living conditions, vector exposure, and preventive care in epidemiological studies.

In Sri Lanka, there are many previous studies on detecting *Dirofilaria* parasites only using the *Dirofilaria*‐specific primers [25, 33, 34] and very limited studies using the species‐specific primers [11]. But there were no samples positive for *D. immiti*s, even though the study had used *D. immitis*‐specific primers [11]. In contrast, the present study used species‐specific primers that could have targeted specific regions of each species and aid in accurate identification of *D. immitis* and *D. repens* that have similar morphological features (Table 8) [35]. Precise identification of the species is really important when multiple species are present in the same blood sample [36].

**Table 8:** Summary table of previous studies and present study recordings on *Dirofilaria* spp. and *Wolbachia*.

Study	Location	Sample size	Diagnostic method	Molecular marker/primer type	Detected species and key findings
Wijetilake et al. [34]	Sri Lanka (unspecified)	Not reported	Microscopy/early parasitological identification	Not applicable	*Dirofilaria* spp. without species differentiation
Nugapola et al. [37]	Sri Lanka	330 mosquitoes	PCR‐based molecular detection	Nonspecies‐specific markers	Detection of *Wolbachia* species associated with mosquitoes
Dasanayake et al. [11]	Kanthale Divisional Secretariat, Trincomalee District, Eastern Province, Sri Lanka	162 canine blood samples	Direct smear microscopy + species‐specific PCR	Species‐specific DIR3/DIR4 primers targeting 5S rRNA gene	58.6% of samples were PCR‐positive for *D. repens*; no *D. immitis* infections were detected
Tharsan et al. [30]	Coastal Jaffna peninsula of northern Sri Lanka	Not reported	Molecular detection	*Wolbachia* surface protein	*Aedes albopictus* were found to be widely infected with the wAlbA and wAlbB strains of *Wolbachia* in Jaffna
Wijegunawardana et al. [38]	Sri Lanka	78 mosquitoes	Molecular detection	wsp and 16S rRNA	*Aedes aegypti* mosquito populations were infected with the *Wolbachia* species
Present study	Colombo District, Sri Lanka	368 canine blood samples	Molecular detection	*Dirofilaria*‐specific primers, *Dirofilaria* species‐specific primers, and *Wolbachia* surface proteins	Detection of *Dirofilaria repens*, *Dirofilaria immitis*, and *Dirofilaria asiatica*. Identification of coinfection of *D. repens* and *D. immitis* in the same host. Detection of the endosymbiont of *Wolbachia* and *Dirofilaria* spp.

The presence of both *D. repens* and *D. immitis* in the same sample in the present study shows the coexistence of species in the same host. In multiple studies, the coexistence of *D. repens* and *D. immitis* has been documented globally. The natural coexistence of *D. repens* and *D. immitis* in dogs in Romania was identified by species‐specific PCR, targeting genes 12S rDNA and the cox1 genes [39]. The same study has conducted a multiplex PCR, but it has failed to amplify the *D. repens* due to *D. immitis* being dominant, whereas a single PCR confirmed the presence of both species in the same sample [39]. Another case report from Poland also confirmed mixed infections in the dog′s blood, confirming that even in regions not traditionally considered endemic, both species can coexist in one individual host [40].

Following the molecular confirmation of *Dirofilaria* species through optimized PCR conditions, phylogenetic analysis was also performed to identify the genetic relationships of the obtained sequence with previously reported sequences. The sample (Accession Number: PX945309) isolate clustered tightly within the *D. immitis* clade, showing a very close relationship with multiple references to *D. immitis*. The genetic distance values between the samples and the reference sequences were about ~0.00–0.02, which indicates the minimal sequence divergence. According to study results, the close genetic similarity observed suggests a conserved *D. immitis* population in Sri Lanka. Previous studies that have been demonstrated in Sri Lanka indicate that the Sri Lankan *D. immitis* could form a well‐supported monophylogenetic clade with global *D. immitis* sequences [26, 41].

This study′s findings reveal the endosymbiont of *Wolbachia*–*Dirofilaria* in Sri Lanka as the first record. These findings can provide additional insight into the parasite–host–microbe interactions [42, 43]. According to the studies that have been conducted, *Wolbachia* is strongly associated with *D. immitis* infections. The bacteria show a strong endosymbiosis with *D. immitis*, as it is essential for the parasite biology, such as nutrient provisioning, embryogenesis, and development [27, 43, 44]. Interestingly, according to the findings of the present study, the *Wolbachia* showed endosymbiosis with *D. asiatica* as well. A molecular study that has been conducted in Thailand has discovered *Wolbachia* in *D. asiatica* infections and characterized the bacteria into supergroups C and F, which normally infect the filarial nematodes [45].

In the present study, *Wolbachia* DNA was specifically confirmed to be positive for *Dirofilaria* spp. using species‐specific PCR assays. This sequential detection approach strengthens the likelihood that the identified *Wolbachia* DNA was biologically associated with *Dirofilaria* parasites rather than originating from free‐living or unrelated bacterial sources ([36, 46]. Therefore, the detection of *Wolbachia* DNA exclusively in *Dirofilaria*‐positive samples in this study is consistent with the known endosymbiotic relationship between these organisms. In order for definitive confirmation of the endosymbiont of *Wolbachia* with *Dirofilaria* spp. would require visualization of *Wolbachia* within *Dirofilaria* tissues using techniques such as fluorescence in situ hybridization (FISH). Nevertheless, given the obligate and highly conserved association between *Wolbachia* and filarial nematodes, the findings of this study strongly suggest that the detected *Wolbachia* DNA is derived from endosymbiotic bacteria residing within the *Dirofilaria* parasites.

In the present study, one sample was detected with both *Wolbachia* and the coexistence of two *Dirofilaria* species: *D. repens* and *D. immitis*. This endosymbiosis, as well as the coexistence, shows the complex biological relationships between nematodes and the bacteria. Global research has documented the presence of the *Wolbachia* bacteria in *D. immitis* and *D. repens* at approximately 30% and 52%, suggesting endosymbiosis [47]. From the perspective of clinical and epidemiological studies, the detection of *Wolbachia* in filarial infections with multiple species has many implications [48, 49]. According to literature, confusion could happen due to mixed or coinfection or low‐level filarial infections or due to the environmental or vector‐related *Wolbachia* signals, especially when the wsp gene is used [50, 51].

The phylogenetic analysis of the *Wolbachia* wsp gene sequences obtained from *Dirofilaria*‐positive canine blood samples indicated two distinct evolutionary patterns among the studied isolates, WL2 (PX935691) and WR2 (PX935692). Their placement within the phylogenetic tree showed notable divergence, while clustering with different reference *Wolbachia* lineages rather than forming a single, host‐specific clade. The PX935692 isolate clustered closely with *Wolbachia* endosymbionts of *Brugia pahangi*. According to this grouping, it is suggested that this sample belongs to the classical filarial *Wolbachia* supergroup. This supergroup is typically coevolved with nematode hosts and reveals a relatively conserved evolutionary pattern ([22, 52]. But in PX935691, the isolate showed an unexpected phylogenetic relationship with the *Wolbachia* endosymbiont of *P. longicornis*, which is an arthropod host. This kind of distinct clustering indicates a more divergent evolutionary origin. This suggests that this sample may represent either a genetically distinct strain or a recombinant lineage within the *Wolbachia* diversity [20]. Their separation into different clades can be explained by several factors. For example, these *Wolbachia* are transmitted by horizontal methods while allowing movement between unrelated host species. This can happen due to the transmission through shared mosquito vectors that act as intermediate hosts for *Dirofilaria* spp. [52]. Other than that, the overlap between host species and vector‐mediated transmission dynamics may further influence the strain distribution across different *Dirofilaria* species [22].

Identification of *Wolbachia* in canine *Dirofilaria* infections is important not only for the diagnosis but also for informing control strategies. Since the *Wolbachia* bacteria contribute to the development of nematodes, fertility, and pathology, their presence in the nematode body can be used as a marker to identify the nematode species [28, 43]. In the present world, *Wolbachia* has become an important target for adjunctive therapy for *D. immitis*, which uses doxycycline antibiotics to reduce the function of *Wolbachia* in order to deactivate the heartworm and its transmission [28, 43, 53]. Host–parasite interactions in filarial infections involve complex immunological and biochemical responses that influence disease progression and parasite survival. Previous studies have demonstrated alterations in host immune responses and biochemical parameters following parasitic infections and subsequent treatments. For example, Singh et al. [54] reported significant changes in immune and biochemical markers in infected hosts following antiparasitic treatments. Similarly, another study highlights the dynamic interactions between host immune defenses and parasite survival strategies, including immune modulation and evasion mechanisms [55] . Because of these characteristics, in future studies, identification of *Wolbachia*–*Dirofilaria* is important in order to control them.

Several limitations of the present study should be acknowledged. First, the study used convenience sampling, which may not accurately represent the broader canine population in Colombo District, Sri Lanka. Another limitation was that the presence of positive samples was relatively small, which may reduce the generalizability of the findings and limit broader epidemiological interpretation. Furthermore, when constructing the phylogenetic tree, a limited number of reference sequences were included in the analysis, which may be another limitation. In addition, the identification of *Wolbachia* was based primarily on the wsp gene, which has known limitations due to possible amplification bias, mixed lineage detection, and phylogenetic inconsistencies in coinfected samples [50, 51]. Therefore, future studies should incorporate multilocus sequence typing (MLST), larger sample sizes, vector investigations, and localization techniques such as FISH in order to identify *Wolbachia* in *Dirofilaria* tissues and confirm their association. The reduced PCR sensitivity in microfilaria‐positive dogs in the present study may be attributed to several biological and technical factors. One possible reason may be the low‐level or intermittent microfilariae in peripheral blood, which can lead to insufficient template DNA for amplification, particularly when parasites are low [33]. Another factor could be the presence of PCR inhibitors in canine blood, such as heme compounds, which can interfere with DNA amplification efficiency. Other than that, genetic variability in target regions among *Dirofilaria* spp. may also affect primer binding efficiency while giving false‐negative results [21]. These types of limitations were recorded from previous studies as well while highlighting that the molecular diagnosis is highly dependent on parasite load, sample quality, and assay sensitivity [13].

Overall, this present study represents the first molecular‐based confirmation of *D. immitis* infection in canine hosts in Sri Lanka and the first report of the *Wolbachia*–*Dirofilaria* endosymbiosis from the country. These novel findings have important effects on the health of canines as well as human and vector‐borne disease control. Not only that, but the detection of *Wolbachia* has a direct relevance to the disease management and the controlling strategies of canine dirofilariasis by targeting the *Wolbachia* bacteria. This study establishes a molecular basis that will be important for future control measures and surveillance targeting canine dirofilariasis in Sri Lanka.

## 5. Conclusions

The present study provides molecular evidence of *Dirofilaria* infections in canine host samples from the Colombo District of Sri Lanka, including the detection of *D. immitis*, *D. repens*, and *D. asiatica* based on sequence analysis. In addition, molecular detection confirmed the presence of *Wolbachia* DNA associated with *Dirofilaria* spp. in the examined samples. The coexistence of *D. immitis* and *D. repens* was observed in a single host within the study population. According to the breed‐wise observations, there was variation in infection detection among dogs, with stray dogs showing higher representation in the positive samples.

Based on comparison with previously published studies, the detection of *D. immitis* and the molecular identification of *Wolbachia* associated with *Dirofilaria* spp. represent the first reported molecular evidence of these findings in Sri Lanka within the limitations of the available literature.

## Author Contributions

N.L.G.: field collection of samples, bioassays and laboratory experimentation, writing the manuscript, and data analysis. K.R.: research design, overall supervision of the project, writing the manuscript, data analysis, and review and editing. W.R.: supervision and review and editing of the manuscript.

## Funding

This study was funded by the United Nations Educational, Scientific and Cultural Organization (10.13039/100005243) (TWAS Research Grant 24‐423 RG/BIO/AS_1).

## Disclosure

All authors read and approved the final manuscript.

## Ethics Statement

Ethical approval was obtained from the Institute of Biology (IOB), Sri Lanka, under ERC IOBSL 413/03/2025.

## Conflicts of Interest

The authors declare no conflicts of interest.

## Data Availability

The data that support the findings of this study are available from the corresponding author upon reasonable request.
